# Histological study of chronic pulmonary aspergillosis

**DOI:** 10.1186/s13000-015-0388-8

**Published:** 2015-09-03

**Authors:** Naobumi Tochigi, Takao Ishiwatari, Yoichiro Okubo, Tsunehiro Ando, Minoru Shinozaki, Kyoko Aki, Kyoko Gocho, Yoshinobu Hata, Somay Y. Murayama, Megumi Wakayama, Tetsuo Nemoto, Yasuhiro Hori, Kazutoshi Shibuya

**Affiliations:** Department of Surgical Pathology, Toho University School of Medicine, 6-11-1 Omori-nishi, Ota-ku, Tokyo 143-8541 Japan; Division of Respiratory Medicine, Toho University School of Medicine, 6-11-1 Omori-nishi, Ota-ku, Tokyo 143-8541 Japan; Division of Chest Surgery, Toho University School of Medicine, 6-11-1 Omori-nishi, Ota-ku, Tokyo 143-8541 Japan; Laboratory of Molecular Cell Biology, School of Pharmacy, Nihon University, 7-7-1 Narashinodai, Funabashi-shi, Chiba 274-8555 Japan

## Abstract

**Background:**

Chronic pulmonary aspergillosis (CPA) has been accepted the criteria for the diagnosis of pulmonary *Aspergillus* infection. Whereas, either pathophysiology or signs of CPA remains still controversial.

**Methods:**

In this study, we histopathologically investigated 25 specimens of CPA, surgically resected.

**Results:**

21 (84 %) of that comprised male. There were 21 cases with mild impairment of the immune system and/or a scar mostly due to old tuberculosis. There is a tendency for a negative correlation between peripheral blood white cell numbers and value level of beta-(1,3)-D-glucan. Four cases showed a granular fluorescent signal in granulation tissue surrounding the cavity without the fungal aspects itself.

**Conclusions:**

In conclusion, acute inflammatory exudate along the terminal respiratory tract is most significant pathophysiolocial complication of the CPA, caused to organizing pneumonia, which derives fatal respiratory failure. In addition, the viability of fungus does not concern extension of exudative inflammation at the site of erosion along terminal airway.

## Background

Chronic pulmonary aspergillosis (CPA) has become an accepted criterion for the diagnosis of pulmonary *Aspergillus* infection, whereas the use of other aspects involving pathophysiology or clinico-pathology remains controversial. CPA tends to occur in elderly and/or debilitated individuals who might not otherwise be immunodeficient. The underlying chronic cavitary lung disease may be due to prior tuberculosis, bullous lung disease, chronic interstitial disease, lung irradiation, surgical lung resection, lung infarction, or cystic fibrosis [1]. End-stage sarcoidosis is a common cause of the cystic remodel associated with CPA. Pathophysiology of CPA may be essentially defined by epithelial destruction and localized infiltration of fungi induced by mild impairment of the immune system with airway anatomical reconstruction. Previously, semi-invasive pulmonary aspergillosis [2], chronic necrotizing pulmonary aspergillosis [3], and chronic cavitary pulmonary aspergillosis [4] were suggested clinically. However, there are few histopathological studies of CPA [5]. In this study, we investigated surgically resected CPA specimens histopathologically and analyzed the structure of the pre-existing cavity found in most cases. Additionally, we attempted to clarify the pathogenesis of CPA by analyzing laboratory data from CPA cases, and suggest effective tools to monitor the pathogenesis of CPA.

## Materials and methods

This study was approved by the ethics committee of Toho University (approval number: 2600524051). We reviewed the medical records of Toho University Omori Medical Center from 1999 to 2013, and found 25 surgically resected CPA cases. Firstly, we analyzed the character of the cavity surrounding the fungus ball histopathologically using hematoxylin-eosin double stain (H-E), Grocott’s methenamine silver stain (GMS), and elastic van Gieson stain (EVG). Immunohistochemical staining for cytokeratin was done as a routine procedure. We measured the erosion ratio for each case using a representative slide. Secondly, we collected laboratory data including white blood cells in peripheral blood (WBC), c-reactive protein (CRP), and beta-(1,3)-D-glucan (BD). In the BD assay, we used Fungitec® G test MK (Seikagaku Corporation, Tokyo) or MK-II (Nissui Pharmaceutical Co. Ltd., Tokyo). Thirdly, we analyzed the diffusion of fungal ingredient which can be detected as fine granules by glucan-specific tissue fluorescent assay system (Fungiflora Y®, Trust Medical Co. Ltd., Kasai, Hyogo) at the eroded tissue consisting cavity wall.

## Results

Table 1 summarizes details of the 25 surgically resected CPA cases. The age of patients varied from 28 to 78 years (median 60), 21 cases (84 %) were male, and all cases involved an upper lobe lesion. There was no severe impairment of the immune system, such as malignant hematopoietic tumor or cytotoxic chemotherapy. However, mild impairment of the immune system occurred by a low dose of cortico-steroid administration (SA) and/or diabetes mellitus (DM). Additionally, a scar mostly due to old tuberculosis (OT) was recorded in nine cases. There were 21 cases (84 %) with SA and/or DM and/or OT.

**Table 1:** Character of 25 surgically resected CPA cases

Age	Sex	Locus	SA	DM	OT	CRP	WBC	BD	ER	SH	EI	CO	GF
28	M	RU				0.2	7.1	12.3	78.3				
37	M	RU	Y			0.4	5.4	N/A	100.0				
39	F	LU	Y			15.5	14.0	11.0	100.0			Y	Y
43	F	LU				2.3	5.7	N/A	100.0				
43	M	RU			Y	7.9	7.6	9.3	32.7		Y		
43	M	LU	Y			0.3	11.8	23.9	11.4	Y			
50	M	RU		Y		0.1	10.6	5.0	100.0	Y	Y		
55	M	LU		Y	Y	0.5	5.7	492.0	46.7	Y			Y
56	F	RU			Y	0.2	4.9	8.7	46.2	Y			
57	M	RU		Y	Y	1.0	5.8	N/A	42.9	Y	Y		
58	M	LU			Y	0.2	3.1	67.6	44.7	Y		Y	Y
59	M	RU		Y		0.2	13.5	N/A	100.0		Y		
60	M	RU	Y			0.7	10.7	5.0	57.9				
61	M	RU	Y	Y		0.3	9.9	27.7	54.5	Y			
61	M	LU				0.6	6.2	6.1	3.7	Y	Y		
64	M	RU			Y	0.1	5.1	18.2	38.7				
66	M	LU				6.6	9.3	5.0	75.5	Y	Y		
67	F	LU			Y	1.4	6.7	24.2	79.3	Y			
68	M	RU		Y		0.7	9.3	5.0	81.1		Y	Y	
69	M	LU	Y			7.9	6.5	11.4	3.8				
71	M	RU		Y	Y	2.0	6.7	5.2	85.4	Y	Y		
73	M	RU				5.4	8.2	N/A	88.6	Y		Y	Y
74	M	RU		Y		0.2	4.0	29.8	95.3				
75	M	LU		Y	Y	1.9	4.2	7.1	12.9	Y			
78	M	RU		Y		4.8	7.5	10.7	69.2		Y		

*RU* right upper lobe, *LU* left upper lobe, *SA* history of cortico-steroid administration, *DM* diabetes mellitus, *OT* scar mostly due to old tuberculosis, *CRP* c-reactive protein, *WBC* white blood cells in peripheral blood, *BD* beta-(1, 3)-D-glucan, *ER* erosion ratio (%), *SH* Splendore-Hoeppli phenomenon, *EI* eosinophil infiltration, *CO* calcium oxalate crystal deposition, *GF* granular fluorescent signal at granulation tissue surround the cavity, but not identified fugal aspects

**Fig. 1:**
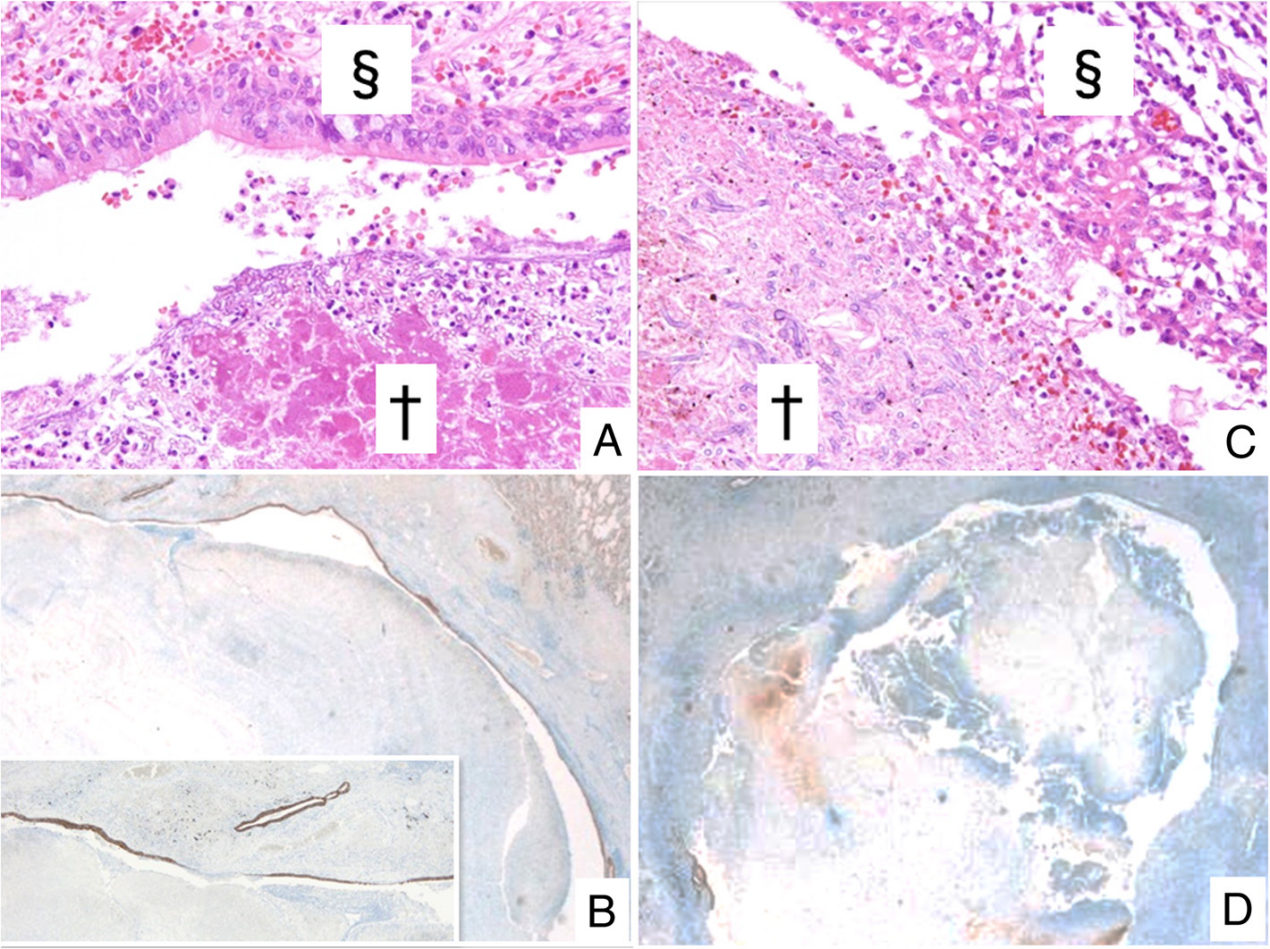
Histopathology of erosion caused by CPA. **a** Cavity [§] is covered by ciliated epithelium without erosion (H-E). Note the Splendore-Hoeppli phenomenon (eosinophilic staining in fungus ball [†]). **b** Immounohistochemical staining for cytokeratin AE1/AE3 is done in case A. Erosion ratio is 3.7 %. **c** Epitheliums disappear and inflammatory granulation tissue exposes the surface of cavity. **d** Immounohistochemical staining for cytokeratin AE1/AE3 is done in case A. Erosion ratio is 95.3 %

**Fig. 2:**
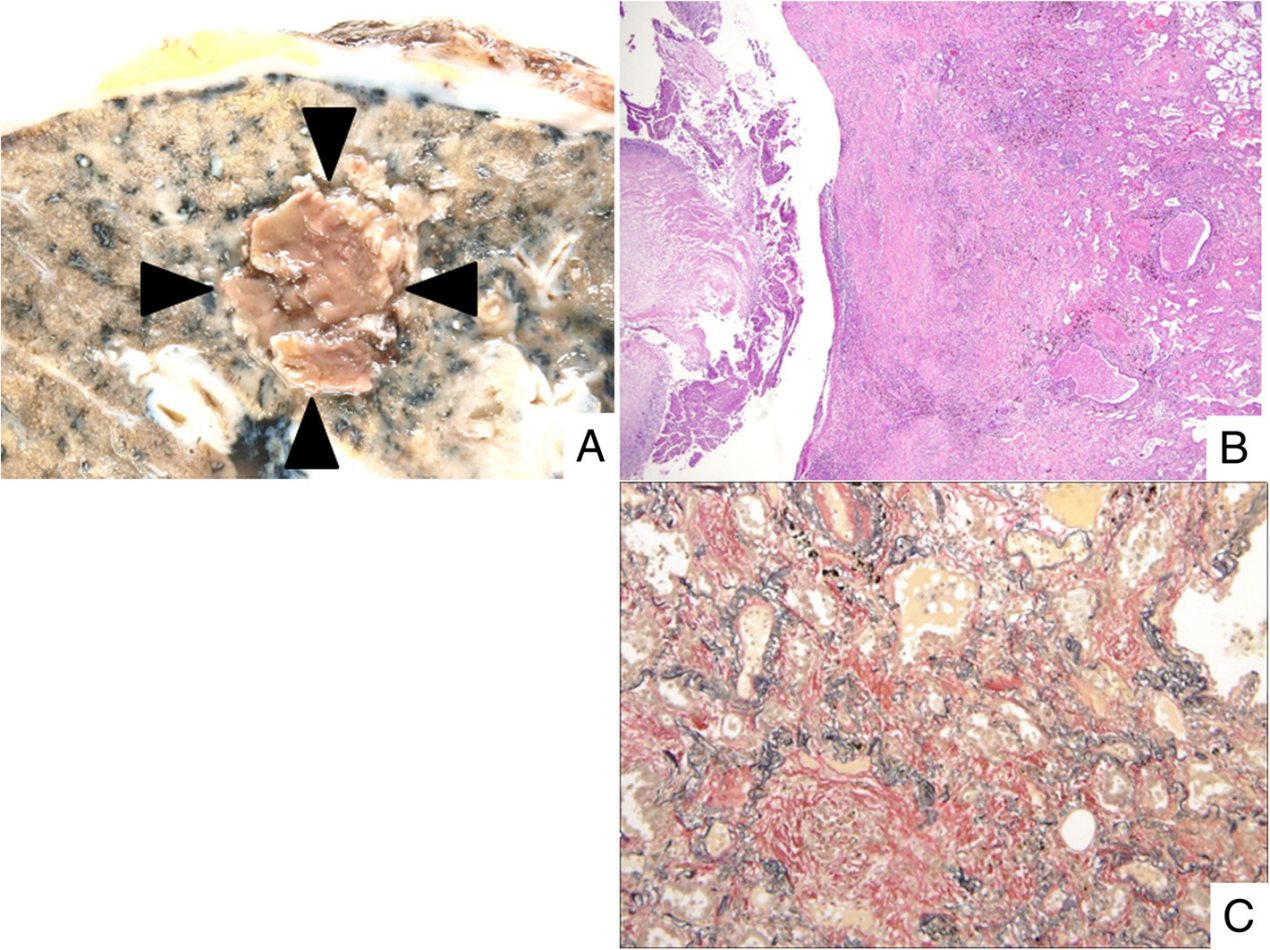
Histopathology of organization surrounding the cavity. **a** Macroscopic findings reveal the fungus ball (arrow head). Pleura with fibrous thickening is also noted. **b** Organization area can be seen around the fungus ball (H-E staining). **c** Alveolar spaces are filled with dense collagenous tissue (EVG staining)

Histopathological examination revealed erosion of the cavity surrounding the fungus ball in all 25 cases. Nine cases exhibited eosinophil infiltration clearly. No epithelioid cell granuloma was detected. The erosion ratio was 3.7–100 % (mean: 62.0 %). The mean erosion ratio with the Splendore-Hoeppli phenomenon (SH) was 53.2 %, otherwise it was 71.4 % without SH (Fig. 1). Interestingly, there was a wide organization area surrounding the cavity without fungal aspects in some cases (Fig. 2). This suggests that we cannot explain this phenomenon using conventional infectious theory which involves local proliferation of microorganisms.

**Fig. 3:**
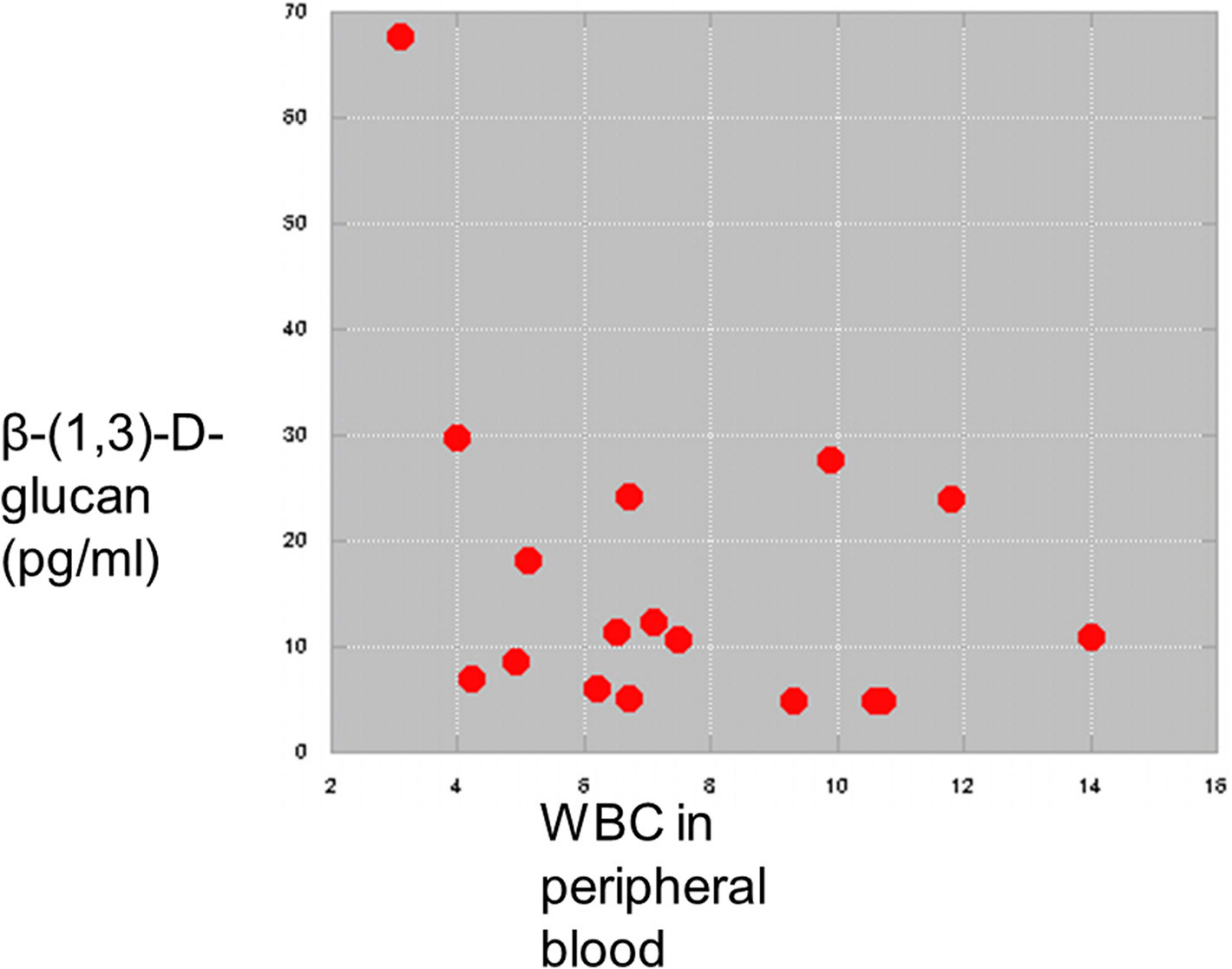
Correlations between WBC and BD

A correlation between WBC and BD is shown in Fig. 3. There is a tendency for a negative correlation between WBC and BD, although it was not statistically significant. These data suggest that impairment of the immune system worsens CPA pathogenesis.

**Fig. 4:**
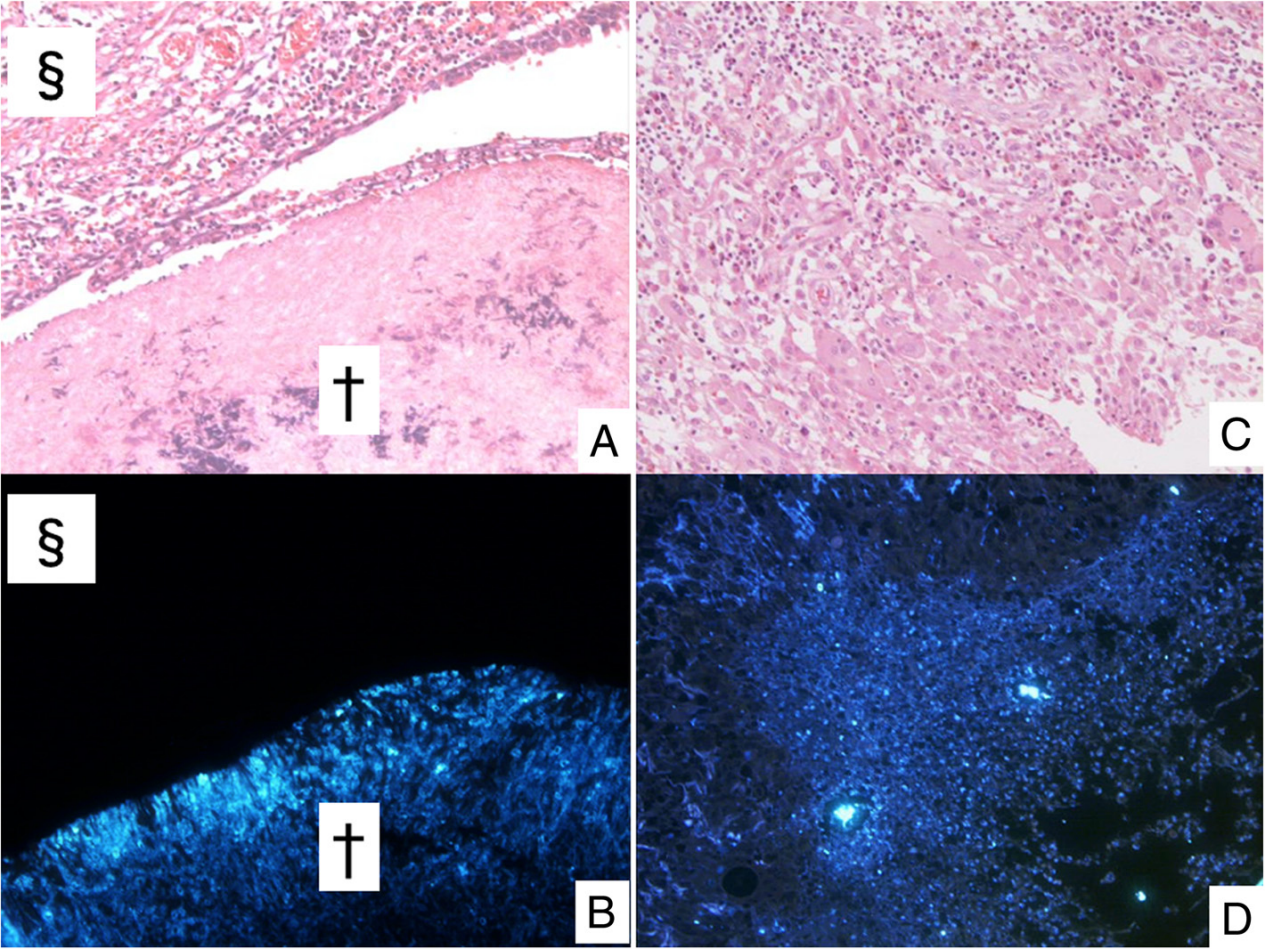
**a** Fungus ball (†) and surrounded cavity (§) can be seen (H-E staining). **b** Positive signal can be detected only in fungus ball using Fungiflora Y®. **c** There is erosive area without fungal aspects (H-E staining). **d** Granular fluorescent signal at granulation tissue surrounded the cavity

Four cases (16 %) showed a granular fluorescent signal in granulation tissue surrounding the cavity using Fungiflora Y® stain, but we did not identify fungal aspects (GF) using H-E or GMS (Fig. 4). Among the 19 cases with measured BD before the surgical resection, the three GF-positive cases had a BD of 190.2 ± 201.2 pg/ml, while the 16 GF-negative cases had a BD of 12.8 ± 7.5 pg/ml. The detected correlation between the presence of GF and the high level of BD is statistically significant (one-sided test, *p* = 0.027, Mann–Whitney *U* test).

## Discussion

Gefter et al. defined semi-invasive pulmonary aspergillosis as a chronic cavitary form of pulmonary aspergillosis with mild immunosuppression or underlying lung disease [2]. They indicated that this form of aspergillosis was part of a spectrum of disease ranging from saprophytic to frankly invasive types. This was followed by report from Binder et al. which finally defined chronic necrotizing pulmonary aspergillosis [3]. Within recent decade, Denning et al. described chronic cavitary pulmonary aspergillosis and chronic fibrosing pulmonary aspergillosis using radiological findings [4]. On the other hand, because of the difficulty to distinguish chronic necrotizing pulmonary aspergillosis and chronic cavitary pulmonary aspergillosis, Izumikawa et al. proposed chronic progressive pulmonary aspergillosis, which included chronic pulmonary aspergillosis of both type; necrotizing and cavitation [6]. However, there are few reports conducting histological and pathophysiological analyses on this chronic form of aspergillosis with reference to some representative monitoring system. Whereas our literature search for previous study could highlight the best-constructed histopathological study of Yousem reviewed 10 CPA cases and yielded a classification with three categories of granulomatous response [5], little were discussed on the pathophysiology of the disease. With comparison to Yousem’s subject group, there might be closer relationship between sequela of tuberculosis and CPA emerged from our study which can be supported by a result that our 25 CPA cases involved an upper lobe that the commonest area of the primary lesion of tuberculosis. However, since none of our patient showed active granuloma that must be essentially induced by tuberculous infection, previous tuberculous infection might simply played a role to re-construct airway, and an active tuberculous infection has little contribution to develop the pulmonary lesion.

Some our CPA cases showed pre-existing airway re-modeling, such as cavitary formation, most of which might be sequel to old tuberculosis followed by emphysema. This anatomical reconstruction can promote infestation or saprophytic proliferation of mold onto the lining epithelial cells of the altered airway. Whereas saprophytic proliferation of mold itself may irritate lining epithelial cells to occur erosion, mild impairment of the immune system indexed by steroid administration and/or diabetes mellitus in our study can play an important role to induce active state of erosion or ulcer accompanied with massive exudate. There was little hyphal component in inflammatory exudate filling the alveolar space even that has no direct connection to altered large air spaces containing molds. Therefore, organization associated with CPA usually led severe respiratory failure can be understood as result of spreading of acute inflammatory exudate provided from active erosion of the altered airway, mostly corresponding to cavity, but progression of organization was not concerned whether the exudate containing fungal element or not. Another analysis of our study revealed diffusion of fungal components at the organizing area around the cavity wall, which could be detected as fine fluorescent granules by Fungiflora Y® system. Consequently, We wish to emphasize that the organizing pneumonia known as a serious complication of the CPA is induced by spreading of acute inflammatory exudate via airway, and this phenomena has much lesser concerning whether mold is viable or not. On the other hand, whereas BD testing has been accepted as one of the useful tool for diagnosis of invasive aspergillosis, of which value is reflecting extent of fungal exposure to the blood flow, implication of BD test has still been controversial in case of CPA. A part of this may be explained that mold intermingled with inflammatory exudate in the cavity is isolated from the patient’s blood flow with various degree by the altered airway usually consisted with inflammatory granulation tissue and fibrous tissue including neutrophils, mononuclear cells, and fibroblasts. In the present study, we found a negative correlation between WBC and BD, although it was not statistically significant, which suggests that neutrophil activity not only kill the invading fungi, but also prevent to transfer BD from feeble fungi into blood flow, since the main defense against mold is the action of neutrophils. As described above, some CPA cases showed an organization area without a fungal body. In addition, we detected a granular fluorescent signal in inflammatory granulation tissue surrounding the cavity in 4 cases (16 %) without fungal aspects (GF). Among these cases, which had a measured BD before the surgical resection, we found a statistically significant association between a high level of BD and the presence of GF. Therefore, an increasing of BD in case of CPA can be understood as a sequel to widely spread diffusion of fungal components largely consisted with BD. There may be much lesser contribution to increase BD that the direct penetration of fungi into the tissue or blood flow, of which situation is usually confirmed in case of invasive aspergillosis with agranulocytosis [7].

## Conclusions

We wish to conclude that extension of acute inflammatory exudate along the terminal respiratory tract is most significant pathophysiological complication of the CPA, because the spreading of exudate filling alveolar space must cause organizing pneumonia, which derives fatal respiratory failure. In addition, the viability commonly suggesting the invasiveness of saprophytic *Aspergilli* does not concern progression of exudative inflammation at the site of erosion.
